# Multifunctional Janus Hydrogels: Surface Design Strategies for Next-Generation Clinical Solutions

**DOI:** 10.3390/gels11050343

**Published:** 2025-05-06

**Authors:** Taoxu Yan, Junyao Cheng, Haoming Liu, Yifan Wang, Chuyue Zhang, Da Huang, Jianheng Liu, Zheng Wang

**Affiliations:** 1 Department of Orthopedics, Chinese PLA General Hospital, Beijing 100853, China; yantaoxu0409@163.com (T.Y.); cjyspine@163.com (J.C.); haomingliu325@163.com (H.L.); wyfspine@163.com (Y.W.); cycycyzhang301@163.com (C.Z.); 2 College of Biological Science and Engineering, Fuzhou University, Fuzhou 350108, China; huangda@fzu.edu.cn

**Keywords:** Janus hydrogels, tissue engineering, hemostasis, smart medical systems, orthopedic repair

## Abstract

Janus hydrogels, distinguished by their dual-sided structure with distinct physical and chemical properties, have garnered significant attention in the medical field, particularly for applications in drug delivery, tissue engineering, and wound healing. Their ability to simultaneously perform multiple functions, such as targeted drug release and biomimetic tissue interaction, positions them as a promising platform for advanced therapeutic strategies. The growing interest in these hydrogels is primarily driven by their multifunctionality and capacity to address complex biological needs. This review delves into the design, fabrication methods, and applications of Janus hydrogels in medicine, focusing on their potential to overcome the limitations of conventional therapies and providing a comprehensive overview of their role in contemporary biomedical applications.

## 1. Introduction

Janus hydrogels are emerging multifunctional soft materials characterized by two distinct yet integrated regions within a single continuous network. In contrast to conventional hydrogels—which are structurally homogeneous and exhibit symmetric properties—Janus hydrogels possess a biphasic architecture with asymmetric composition or surface chemistry on each “face” [1,2,3]. Such an internal partitioning can be realized, for example, by designing one side of the hydrogel to be highly hydrophilic, while the opposite side is less hydrophilic or even hydrophobic. This unique two-faced structure endows Janus hydrogels with anisotropic physicochemical properties and interactions unattainable in single-network hydrogels. Unlike a uniform hydrogel, which interacts with its environment uniformly, a Janus hydrogel can simultaneously present distinct interfaces—each tailored to interact with different substances or phases—thereby mimicking natural heterogeneous interfaces in ways conventional hydrogels cannot [4,5,6]. The result is a material platform that combines disparate functionalities within one hydrogel, setting Janus hydrogels apart as a fundamentally new class of designer hydrogels [7,8].

Building on this asymmetric architecture, Janus hydrogels offer several key advantages over traditional and even other advanced hydrogels. One major advantage is their directional functionality: each side or region can be independently engineered to perform a specific function, allowing the hydrogel to carry out multiple roles in a spatially segregated manner [9,10,11]. For instance, in a bioadhesive Janus hydrogel, the basal surface can be chemically tailored for strong, instant adhesion to a target tissue, while the apical surface is formulated to resist adhesion. This dually functional surface design effectively provides opposing interactions on each side, a feat that mitigates problems like unwanted tissue adhesion or biofouling that often occur with symmetric hydrogel implants. Janus hydrogels also exhibit enhanced interfacial reactivity and compatibility [12,13]. As they present two different chemistries simultaneously, they can mediate interactions between disparate environments more effectively than single-phase hydrogels [14,15].

Furthermore, Janus hydrogels can be designed to provide spatially resolved responsiveness to external stimuli. As one region can contain stimuli-sensitive moieties (photothermal agents, pH-responsive polymers, magnetic nanoparticles, etc.) and the other region can remain inert or sensitive to a different stimulus, the material can produce anisotropic responses [16,17]. For example, an asymmetric hydrogel actuator might bend or shape-shift in one preferred direction when triggered, as only one side swells or contracts, while the other side provides a restraint. In general, this compartmentalized reaction to stimuli enables complex behaviors—such as directional deformation, sequential drug release, or gradient signal propagation—that are beyond the scope of conventional hydrogels [18,19].

Here are some fabrication approaches for Janus hydrogels. The “one-pot” strategy with external stimuli creates asymmetry during hydrogel formation by applying external forces, such as magnetic fields, centrifugation, or temperature gradients. For example, magnetic Fe_3_O_4_ nanoparticles are concentrated on one side under a magnetic field, while hydrophilic and hydrophobic components separate through stirring or buoyancy. Components self-segregate into distinct layers or gradients based on differences in density, solubility, or responsiveness to stimuli. This approach enables dual functionalities, such as photothermal activity on the Fe_3_O_4_-enriched side and mechanical flexibility on the other side. It is particularly efficient for producing bulk Janus hydrogels with intrinsic asymmetry, such as hydrogels that adhere to tissues on one side and resist adhesion on the other.

Unilateral-layer modification involves post-synthesis treatment of one surface of a preformed hydrogel through chemical grafting, spray coating, or physical deposition. For instance, spraying hydrophobic fluorinated agents or grafting chitosan can create surfaces with anti-adhesive or antibacterial properties. Asymmetry is introduced through surface treatment without altering the bulk structure of the hydrogel. This method allows the tailoring of specific surface functionalities, such as introducing a conductive Ag nanowire layer for sensing applications or a zwitterionic polymer layer to prevent protein adhesion, offering precise control over surface interactions.

Dual-layer integration combines two distinct hydrogel layers, such as adhesive polyacrylamide and non-adhesive alginate, using sequential casting, 3D printing, or electrostatic bonding techniques. Physically distinct layers with independent properties, such as mechanical strength, porosity, or drug release profiles, are integrated into a single construct. This method combines complementary roles, such as providing a soft, cell-adhesive layer for tissue integration and a rigid, anti-fouling layer for structural support, making it suitable for applications like wound dressings or multifunctional sensors.

In terms of application impact, the “one-pot” method is ideal for the scalable production of hydrogels with intrinsic asymmetry, such as those used in solar steam generation. Unilateral modification enables targeted surface functionalities, such as antibacterial coatings for medical devices. Dual-layer integration supports complex designs like core-shell structures for controlled drug delivery or wearable sensors with anisotropic conductivity. Each fabrication approach leverages distinct mechanisms to achieve asymmetry, directly influencing the hydrogel’s biomedical, environmental, or energy-related functionalities.

Notably, the synergistic coupling between the two distinct sides can even amplify material performance. Anisotropic or layered hydrogel architectures have been shown to achieve superior mechanical and thermal properties relative to uniform networks; for instance, a recent study demonstrated that a layer-by-layer structured hydrogel attained remarkable flame retardancy and thermal stability (a limiting oxygen index of ~83.5%), along with high mechanical strength, outperforming various single-component biomass hydrogels. Such findings underscore that the Janus configuration is not only about combining two functions but also leveraging synergy at the interface of the two phases to create emergent properties [20,21].

In orthopedic and musculoskeletal reconstruction, Janus hydrogels function as critical support materials, aiding in the repair of damaged bones and soft tissues. Their dual-layer structure ensures vital mechanical support and excellent biocompatibility, thus promoting bone healing while effectively reducing inflammation. In the realm of oral and maxillofacial repair, the hydrogels’ tunable wettability offers robust adhesion while preventing undesired tissue attachment, contributing to improved recovery outcomes for patients [22,23,24]. Furthermore, the advanced functionalities of Janus hydrogels enable them to respond to various external stimuli, such as temperature, pH, or light, providing precise regulation of drug release and tissue repair processes [25,26]. In intelligent drug delivery applications, they facilitate the targeted, condition-dependent release of therapeutic agents, greatly enhancing treatment efficacy while minimizing unwanted side effects [27,28,29].

**Figure 1:**
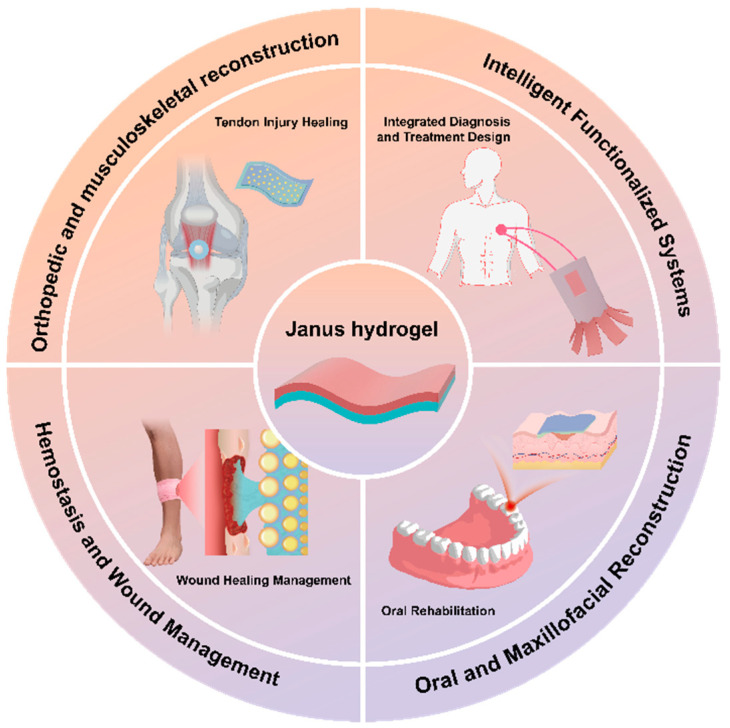
Clinical applications of Janus hydrogels.

In summary, Janus hydrogels distinguish themselves from conventional hydrogels by integrating two spatially segregated functionalities within a single material system, thereby achieving superior and, in certain domains, even breakthrough performances. Their amphiphilicity and anisotropy represent only surface-level advantages. Their deeper value lies in three core attributes derived from their dual-structured design: directional functional targeting, synergistic interfacial interactions, and region-specific responsiveness. These properties empower Janus hydrogels to address complex biomedical challenges—such as dual-mode dressings that simultaneously achieve strong adhesion and anti-adhesion to tissues, intelligent bone repair materials that promote osteointegration while mitigating stress shielding, and programmable minimally invasive delivery devices—applications that pose fundamental obstacles to traditional homogeneous hydrogels. With the field experiencing exponential growth and progressive breakthroughs in key technological bottlenecks, a systematic review of Janus hydrogel research progress holds significant academic value. This article comprehensively analyzes molecular design strategies and interfacial engineering principles, elucidates their distinctive physicochemical behaviors compared to conventional materials, and highlights their cutting-edge biomedical applications in wound management, bone tissue engineering, and beyond (Figure 1).

## 2. Hemostasis and Wound Management

### 2.1. Hemostatic Mechanisms and Interface Design Advantages

Efficient and prompt hemostasis plays a pivotal role in enhancing patient outcomes and safeguarding lives, particularly in critical settings such as emergency care, surgical procedures, and both civilian and military environments. Conventional hemostatic materials often fall short of effectively managing severe, uncontrolled bleeding. This limitation has driven significant interest in advancing the development of more innovative and reliable hemostatic solutions [30,31].

Janus hydrogels stand out for their unique hemostatic mechanisms and superior interface design, making them highly effective in promoting wound healing. Their mechanism centers on the hydrogel’s asymmetric configuration, which cleverly combines adhesive and non-adhesive features [32]. The adhesive layer facilitates rapid blood clot formation by binding to the wound site, thus providing immediate hemostasis. In contrast, the non-adhesive layer prevents unwanted tissue adherence, minimizing the risk of complications such as scarring and tissue damage, which are common in post-surgical recovery. This dual-layer structure enhances the gel’s functionality, making it a promising material for more advanced, controlled wound care [33,34,35].

**Figure 2:**
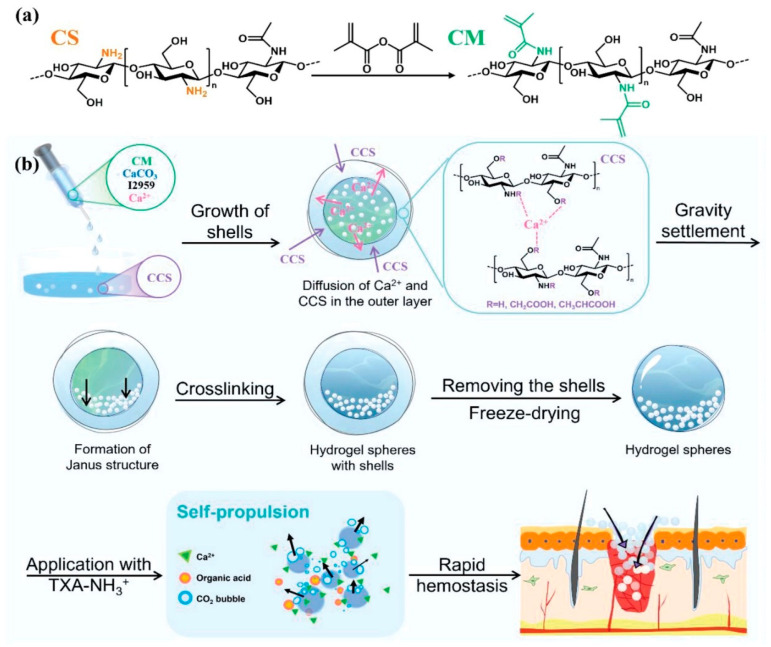
Application of Janus hydrogels in hemostasis. (**a**) Scheme for the synthesis of CM. (**b**) Schematic illustrations for the preparation and hemostatic application of the J-CMH@CaCO3/T. Copyright 2022 WILEY ADVANCED [36].

The authors engineered a self-propelling Janus-structured chitosan-based hydrogel microsphere (J-CMH@CaCO_3_/T) incorporating calcium carbonate and tranexamic acid derivatives (TXA-NH_3_^+^), demonstrating substantially enhanced hemostatic efficacy for irregular wounds and non-compressible hemorrhages through synergistic bubble-propelled locomotion and calcium-mediated coagulation (Figure 2). The asymmetric architecture arises from gravitationally induced CaCO_3_ particle sedimentation during synthesis, with subsequent photopolymerization immobilizing this gradient distribution. Intramicrosphere reactions between CaCO_3_ and protonated TXA-NH_3_^+^ generate CO_2_ microbubbles predominantly on the CaCO_3_-enriched hemisphere, creating directional thrust that propels the microsphere against bloodstreams to penetrate deep wound cavities. Concurrently, accelerated Ca^2+^ liberation activates platelet aggregation and fibrin formation, while deprotonated TXA-NH_2_ exerts antifibrinolytic effects, establishing a dual coagulation reinforcement mechanism. The CaCO_3_-dense hemisphere facilitates rapid ionic release, whereas the opposing hydrophilic hemisphere concentrates erythrocytes and platelets through fluid absorption. Compared to traditional hemostats, this system enables rapid hemorrhage control without external compression, exhibits superior adaptability to complex anatomical wounds, and shows particular promise for emergency management of traumatic injuries in combat or accident scenarios, especially in parenchymal organ damage [36].

Sun et al. engineered a Janus-type hydrogel composite (HGO-C), exhibiting differential adhesion characteristics, constructed exclusively from biocompatible natural polymers to address hemorrhage control, microbial colonization, and tissue repair. This bidirectional functionality emerges from the precisely engineered combination of a tissue-adherent substrate (HGO) and a non-adherent overlayer (CGC). Fabrication of the adhesive stratum involved Schiff base crosslinking between Tris-functionalized hyaluronate derivatives and dextran-oxidized gelatin networks, achieving durable bioadhesion through coordinated hydrogen bonds, charge-based attractions, and stable covalent attachments to biological amine/hydroxyl-rich surfaces.

The non-adherent stratum, composed of chitosan-gelatin matrices reinforced with carboxylated nanocellulose, was subjected to calcium chloride treatment, a process generating calcium–carboxyl coordination bridges that establish a biofluid-repellent interface. Interfacial bonding robustness between layers was optimized through staged fabrication protocols and complementary electrostatic/covalent linkage strategies.

Notably, the HGO component’s diverse functional moieties (hydroxyl, carbonyl, and aldehyde groups) facilitate bimodal attachment mechanisms: transient physicochemical associations and permanent covalent linkages with extracellular matrix components. Although existing synthetic Janus constructs attain surface asymmetry, this biomaterial platform demonstrates enhanced host integration through progressive biodegradation and manufacturing versatility, attributes that potentiate its clinical utility in dynamic wound environments requiring simultaneous sealant action and prevention of secondary tissue damage. The design paradigm shifts focus toward leveraging natural polymers’ inherent molecular recognition capabilities rather than relying on synthetic chemistry to impose asymmetric properties [24].

Inspired by the nest-building and edible nest creation of Collocalia birds, Hui et al. developed a Janus composite nanofiber membrane by depositing large self-gelling powder particles (PAA/PEI-CMC) onto nanofibers along a rotational flight path [37]. One side of the material is hydrophilic, facilitating rapid hemostasis and promoting wound healing; the opposite side is hydrophobic, providing antibacterial protection to prevent infection. This design successfully addresses the challenge of hydrogel and hemostatic powder displacement under high-pressure bleeding or thrombosis risk. The hemostatic lower layer, composed of AA/PEI-CMC, exhibits strong adhesive and clotting properties. During the EBS process for producing the PCL nanofibers of the upper layer, a powder blower disperses PAA/PEI-CMC powder into the flight path of the PCL nanofibers, achieving in-air electrostatic adhesion of the two components. The hydrophilic side can complete clotting within one minute without wound preparation, while the hydrophobic side enhances bacterial isolation and improves the sealing capability over wounds through its hydrophobicity.

### 2.2. Expansion of Postoperative Anti-Adhesion Functionality

Postoperative adhesions refer to the abnormal formation of fibrous tissue that connects surgical wounds to surrounding tissues, frequently occurring in areas such as the abdomen, uterine cavity, and spinal region [28,38]. These adhesions can result in severe complications, including dysfunction of organs, persistent pain, bowel obstruction, and infertility, all of which severely diminish the quality of life for affected patients. The development of adhesions is closely associated with factors such as surgical technique, duration of the procedure, and postoperative inflammation. As a result, patients may experience chronic abdominal discomfort, diminished capacity for daily activities, and, in extreme cases, may require additional surgery to address the adhesions. Furthermore, the identification and differentiation of adhesion tissue from normal anatomical structures can present significant difficulties during secondary surgeries. Thus, preventing the formation of postoperative adhesions has become a critical area of research in modern surgical practices [39,40].

Janus hydrogels present a promising solution in the prevention of postoperative adhesions, thanks to their unique bilayer structure that integrates both adhesive and non-adhesive properties. The adhesive layer actively fosters wound closure and tissue healing, while the non-adhesive layer serves to prevent the formation of unwanted tissue connections. Advanced material engineering allows the non-adhesive layer to be finely tuned into a low-adhesion barrier, effectively isolating the surgical site from surrounding tissues and significantly reducing the risk of adhesion formation. In addition, Janus hydrogels possess controllable biodegradability and inherent anti-inflammatory properties, offering prolonged protection after surgery, minimizing harmful tissue responses, and promoting natural tissue regeneration. These combined features make Janus hydrogels a highly effective and innovative approach for preventing postoperative adhesions, significantly improving patient recovery and overall quality of life [41,42,43,44].

**Figure 3:**
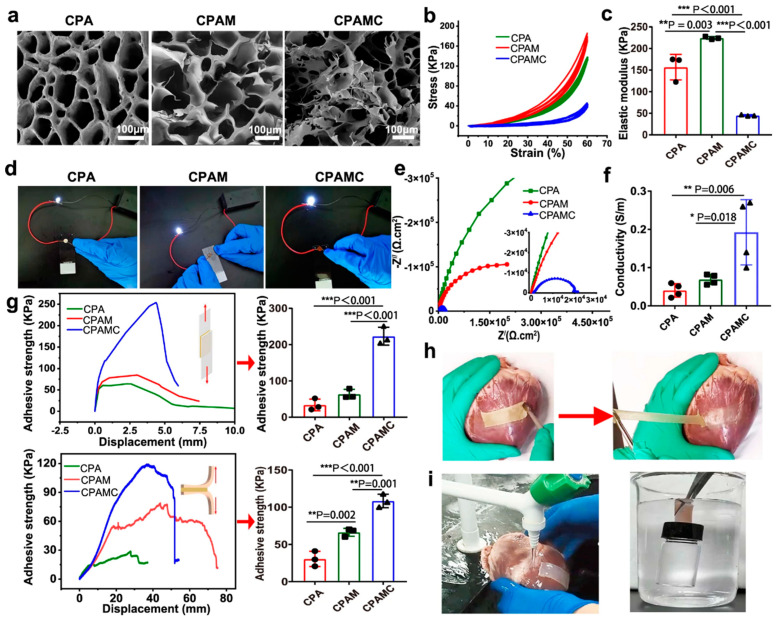
Application of Janus hydrogels in postoperative anti-adhesion functionality. (**a**) SEM images of CPA hydrogel, CPAM hydrogel, and CPAMC hydrogel, scale bars: 100 μm. (**b**) The stress–strain curves of CPA hydrogel, CPAM hydrogel, and CPAMC hydrogel. (**c**) The elastic modulus of CPA hydrogel, CPAM hydrogel, and CPAMC hydrogel (error bar means the standard deviation; ** *p* < 0.01, and *** *p* < 0.001; *p*-values were generated by one-way analysis of variance (ANOVA), followed by Tukey’s multiple-comparison post hoc test; *n* = 3 independent samples). (**d**) The luminance changes of the LED light bulb in CPA hydrogel, CPAM hydrogel, and CPAMC hydrogel. (**e**) Nyquist plots of CPA hydrogel, CPAM hydrogel, and CPAMC hydrogel. (**f**) Statistical analysis of conductivity in CPA hydrogel, CPAM hydrogel, and CPAMC hydrogel (error bar means the standard deviation; * *p* < 0.05, ** *p* < 0.01; *p*-values were generated by one-way analysis of variance (ANOVA), followed by Tukey’s multiple-comparison post hoc test; n  =  4 independent samples). (**g**) Adhesion properties of three hydrogels in dry (glass, upper column) and wet states (muscle tissue of pig heart, bottom) (error bar means the standard deviation; ** *p* < 0.01, and *** *p* < 0.001; *p*-values were generated by one-way analysis of variance (ANOVA), followed by Tukey’s multiple-comparison post hoc test; n  =  3 independent samples). (**h**) Images of CPAMC hydrogel adhering to pig heart. (**i**) Images of CPAMC hydrogel tightly adhered to porcine heart under water flushing (left) and supporting a full-filled bottle under water (right). CAP, CAPM, CPAMC: C, aldehyde cellulose (CNC-CHO); P, polyethylenimine (PEI); A, acrylic acid; M, 3-sulfonic acid propyl methyl acrylic acid potassium (MASEP); C, caffeic acid (CA). Copyright 2022 Springer Nature [45].

He et al. developed the CPAMC/PCA Janus hydrogel, a versatile material aimed at myocardial infarction repair and preventing postoperative adhesions (Figure 3). The hydrogel is structured with an adhesive CPAMC base layer that aids in cardiac recovery. This layer is engineered through a redox-responsive network of aldehyde cellulose (CNC-CHO) and polyethylenimine (PEI), utilizing the polymerization of acrylic acid (AA), 3-sulfonic acid propyl methyl acrylic acid potassium (MASEP), and caffeic acid (CA), which facilitates both covalent crosslinking and non-covalent interactions. On top, the PCA layer consists of AA and carboxylated cellulose (CNC-COOH), crosslinked with polyethylene glycol diacrylate (PEGDA), which provides resistance to cell adhesion and prevents fouling, effectively inhibiting tissue fusion [45].

Unlike the CPAMC hydrogel, the PCA variant lacks catechol, amino, and aldehyde groups but is enriched with carboxyl groups and polyethylene glycol chains. This alteration leads to reduced adhesion properties, significantly enhancing its ability to prevent cell attachment. Thanks to the strong adhesiveness of the CPAMC base layer, the CPAMC/PCA Janus hydrogel can remain securely attached to the heart for prolonged periods, thus preventing adhesion between the heart and chest wall. This hydrogel exhibits remarkable tissue adhesion, elasticity, and conductivity, meeting the specific needs of the myocardial microenvironment for both flexibility and electrical conductivity. Its adhesion characteristics mimic the strain–stress behavior observed in biological tissues and is responsive to glutathione (GSH). Additionally, the research team integrated a complete LED circuit, which demonstrated that the CPAMC hydrogel offers superior conductivity compared to CPA and CPAM hydrogels, as evidenced by the brighter LED emission. The addition of PCA does not interfere with the gel’s cellular conductivity.

Drawing inspiration from the lubricating and adhesive properties found in mussel-derived serum and underwater attachment mechanisms, Li et al. developed a Janus hydrogel with dual asymmetrical adhesion properties. This novel biomaterial overcomes the common limitation of adhesive hydrogels, which often struggle to form strong, stable bonds with tissues and inadvertently contribute to postoperative adhesions due to their inability to prevent separation from surrounding tissues. The hydrogel was synthesized through a stepwise in situ polymerization process. The adhesive layer (AL) is composed of poly(DPAMA-co-SBMA-co-NAGA), offering superior adhesive strength and mechanical stability. By drawing moisture from the tissue surface, the AL layer promotes catechol group adhesion, facilitating effective wet bonding. Additionally, the catecholamine released by the AL layer in vivo helps to decrease reactive oxygen species (ROS) production, thereby supporting wound healing by securing the hydrogel to the tissue and preventing the formation of fibrous gaps between the hydrogel and tissue.

The anti-adhesion layer (AI), constructed from N-acryloylglycylamide (NAGA)-enhanced zwitterionic polymer hydrogels, provides lubricating, anti-fouling, and immune-evasive properties. This layer serves to prevent the formation of postoperative adhesions. In hernia repair animal studies, the Janus hydrogel demonstrated significantly improved wet tissue adhesion, superior anti-adhesion performance, and enhanced tissue regeneration compared to the standard commercially available PP “Mesh” patch [46].

Liu et al. recently developed an amphoteric hydrogel designed to serve as a safe and effective barrier against postoperative adhesions [47]. This hydrogel was synthesized using sulfonic betaine methacrylate (SBMA) as the monomer and *N*,*N*′-methylenebis(2-acrylamide) (MBA) as the crosslinking agent, creating patches with a distinctive hexagonal microstructure. The polymerization process, which employed a free-radical mechanism, involved injecting the prepolymer solution into molds. Drawing inspiration from the surface morphology of a stickleback fish, the hydrogel’s surface features biomimetic grooves that function as microchannels for efficient fluid drainage. This design not only accelerates the interaction between the hexagonal surface and tissue but also enhances the adhesive strength of the hydrogel. When the superhydrophilic amphoteric hydrogel comes into contact with moist surfaces, it absorbs interfacial moisture, while excess water is swiftly expelled through the surface grooves, resulting in a stronger interface bond. Additionally, the independent hexagonal microstructure ensures optimal contact between the material and tissue, further reinforcing the interfacial adhesion, which enhances resistance to detachment and supports sustained functionality during the healing process. The hydrogel’s flat outer surface, characterized by both superhydrophilicity and amphoteric ionic properties, forms a stable hydration layer that prevents protein, bacterial, and cellular adhesion, significantly reducing the risk of postoperative adhesion formation. Furthermore, the hydrogel exhibits remarkable tensile and compressive properties, allowing it to adapt to mechanical stresses induced by organ movements at the surgical site, ensuring its stability within the body throughout the healing process.

## 3. Oral and Maxillofacial Reconstruction

Janus hydrogels offer exceptional adhesion and biocompatibility, enabling them to effectively adapt to the unique conditions of localized environments, which is crucial for tissue repair in the oral and maxillofacial regions. By combining hydrophilic and hydrophobic properties, these hydrogels enhance tissue interaction, facilitate wound healing, and inhibit bacterial proliferation. In oral and maxillofacial reconstruction, Janus hydrogels play a pivotal role in promoting tissue regeneration, preventing infections, and improving overall healing, establishing them as an ideal material for addressing complex injuries and defects in these regions [48,49,50].

Guided bone regeneration (GBR) technology has gained considerable attention as an innovative technique for both horizontal and vertical alveolar bone augmentation, as well as for preserving the alveolar ridge following tooth extraction. A key element of GBR technology is the barrier membrane, which separates soft tissue from the bone defect site. This creates sufficient space for bone regeneration while preventing the overgrowth of soft tissue that could interfere with the process. The success of GBR hinges on four main principles: primary wound closure, angiogenesis, maintenance of osteogenic space, and the stability of both the wound and the implant. Despite the progress, commercially available GBR membranes still face limitations in terms of bioactivity, mechanical adaptability, and biodegradability and fall short in promoting bone and angiogenic regeneration [51,52,53].

Magnesium (Mg) implants have emerged as a promising alternative, offering significant bioactivity, mechanical compatibility, and biodegradability, which eliminate the need for implant removal. However, when the concentration of Mg^2+^ ions exceeds 300 ppm or the pH rises above 9.5, metallic Mg demonstrates antibacterial properties. Yet, the ion concentration in bodily fluids lacks sufficient biological safety and compatibility, and an excessive presence of Mg^2+^ ions can lead to the formation of hydrogen gas. This gas can accumulate in local cavities within the body, adversely affecting both bone healing and vascular reconstruction. Magnesium oxide (MgO), on the other hand, stands out as a multifunctional material for GBR membranes. By releasing additional reactive oxygen species (ROS), MgO nanoparticles (NPs) not only exert antibacterial effects but also alleviate the detrimental impacts of hydrogen gas on tissue growth [54,55].

Wang et al. developed a Janus composite membrane (Mg-MgO/PCL) enhanced with dual forms of magnesium (Mg sheets and MgO NPs) through a combination of casting and electrospinning techniques. The inclusion of Mg sheets within the flexible PCL matrix provided the composite material with the ability to maintain osteogenic space, while the PCL effectively shielded the Mg material from accelerated corrosion due to mechanical deformation. This innovative design addressed the issue of magnesium’s relatively low standard electrode potential (−2.37 V vs. SHE), which often leads to premature implant failure and delayed bone regeneration. The incorporation of MgO NPs enhanced the tensile strength of the Mg-MgO/PCL membrane, reaching 50.3 ± 4.5 MPa. The dense, cast surface of the Janus membrane displayed superior fibroblast barrier properties compared to single-fiber structures. Meanwhile, the porous microfibrous side, mimicking the extracellular matrix and enabling controlled Mg^2+^ release, promoted the adhesion of osteoprogenitor cells, thereby enhancing both osteogenesis and angiogenesis [56].

**Figure 4:**
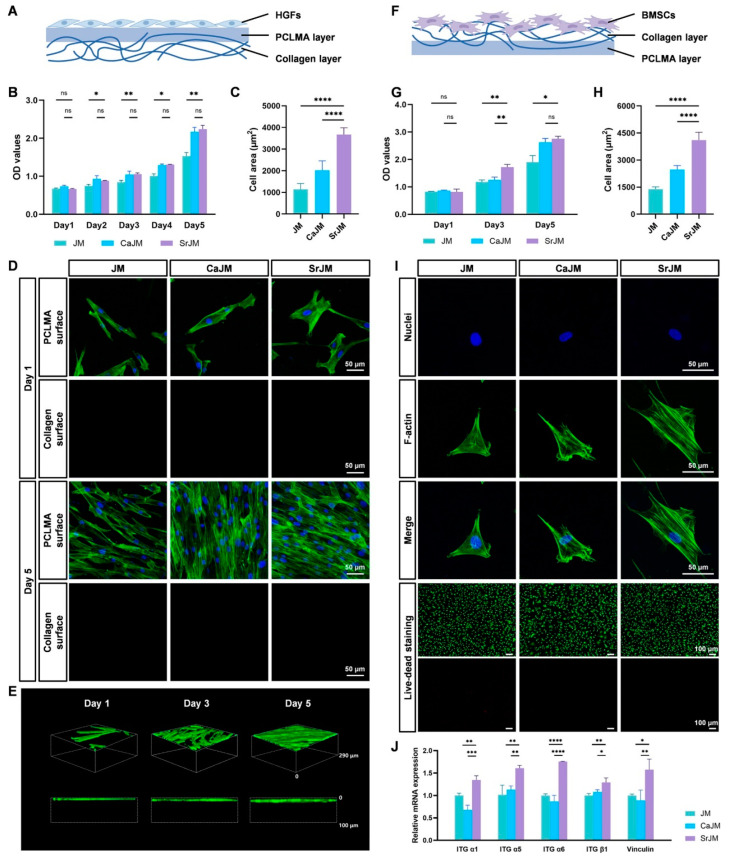
Application of Janus hydrogels in oral and maxillofacial reconstruction. Biocompatibility and barrier function of the Janus membranes. (**A**) Schematic illustration showing that HGFs were seeded on the PCLMA surface of the Janus membranes. (**B**) Proliferation of HGFs cultured on the Janus membranes examined by CCK-8 assay. (**C**) Quantitative results of single-cell area of HGFs. (**D**) Representative fluorescence images of HGFs on PCLMA surface and collagen surface after 1 and 5 days. (**E**) Reconstructed fluorescence images and corresponding cross-section images of HGFs on the PCLMA surface of SrJM. (**F**) Schematic illustration showing that BMSCs were seeded on the collagen surface of the Janus membranes. (**G**) Proliferation of BMSCs cultured on the Janus membranes examined by CCK-8 assay. (**H**) Quantitative results of single-cell area of BMSCs. (**I**) Representative fluorescence images of BMSCs cultured on the Janus membranes for 12 h and live–dead staining assay of BMSCs for 24 h. (**J**) Relative mRNA expression levels of integrin α1, α5, α6, β1, and vinculin in BMSCs for 24 h. ns: *p* ≥ 0.05; *: *p* < 0.05; **: *p* < 0.01; ***: *p* < 0.001; ****: *p* < 0.0001. Copyright 2024 American Chemical Society [57].

Zhao et al. [25] engineered a multifunctional Janus GBR membrane, named SrJM, comprising a porous collagen surface and a dense layer, with the dual purpose of optimizing both osteogenic and barrier functions (Figure 4). The porous collagen surface is specifically designed to enhance bone formation by incorporating amorphous strontium phosphate (ASP) stabilized with polyacrylic acid (PAA) as a precursor for mineralization. This precursor facilitates the internal mineralization of collagen fibers through a liquid-phase process, creating a porous structure that supports cell attachment and promotes osteoblastic differentiation. The dense layer is formed from polycaprolactone methacrylate (PCLMA), which is integral to maintaining the membrane’s barrier function. Photopolymerization was used to coat the PCLMA onto one side of the collagen layer, creating a dense surface that provides mechanical support and prolongs the membrane’s durability. This dense PCLMA layer effectively blocks fibroblast infiltration, while the porous collagen layer encourages the adhesion and osteogenic differentiation of bone marrow-derived mesenchymal stem cells (BMSCs). By activating the Ca^2+^-sensitive receptor (CaSR), integrin, and Wnt signaling pathways, the membrane upregulates osteogenesis-related genes and proteins. Additionally, it promotes the formation of endothelial cell tubes (HUVECs) and stimulates the secretion of angiogenic factors by BMSCs, thereby enhancing angiogenesis. The SrJM membrane also fosters the polarization of macrophages to the M2 phenotype, which produces anti-inflammatory and pro-healing factors that create a favorable environment for bone regeneration. Moreover, the SrJM membrane exhibits excellent biocompatibility with human gingival fibroblasts (HGFs) and BMSCs, promoting both cell adhesion and proliferation. This research not only underscores the osteoinductive benefits of strontium phosphate mineralized collagen but also highlights its significant potential for guiding bone regeneration, providing fresh perspectives for the development of multifunctional GBR membranes [57].

Gingivitis is a persistent inflammatory condition triggered by periodontal pathogens, including *Porphyromonas gingivalis* and *Actinomyces* species, which is the leading cause of damage to periodontal tissues. The primary factor driving periodontal destruction remains bacterial infection. Severe gingivitis, which results in gingival defects, is a global issue, affecting up to 90% of individuals aged 70 and older, imposing a significant economic burden on society. This chronic inflammation can lead to the loss of alveolar bone, tooth loss, and, in more severe cases, systemic inflammation [58,59,60].

Chitosan, known for its remarkable biocompatibility and biodegradability, has limited antimicrobial activity, restricting its applications in tissue engineering. To address this limitation, Huang et al. developed an innovative multifunctional hydrogel based on chitosan, designed to treat periodontitis and stimulate bone regeneration. The membrane is synthesized through a three-step process: (i) Chitosan is dissolved in an alkaline/urea solution and regenerated in an ethanol coagulation bath to form a Janus-structured hydrogel membrane (ChT); (ii) The outer surface is quickly coated with PDA and pSBMA via a CuSO_4_/H_2_O_2_ catalytic system, endowing it with antimicrobial and anti-protein adhesion properties (ChT-PDA-p); and (iii) The inner surface is spin-coated with a gelatin–hydroxyapatite (gelatin-HAP) solution, creating a porous structure that facilitates bone regeneration (ChT-PDA-p-HAP). The outer layer, composed of PDA and pSBMA, prevents bacterial colonization from pathogens like *Escherichia coli*, *Staphylococcus aureus*, and *Porphyromonas gingivalis* while reducing the likelihood of repair failure. Simultaneously, it supports the osteogenic function of the inner gelatin–HAP layer, resulting in a dual therapeutic effect. The co-deposition of PDA and pSBMA significantly enhances the hydrogel’s antimicrobial performance while maintaining chitosan’s biocompatibility and biodegradability. Moreover, using chitosan as a base material not only lowers production costs but also improves the membrane’s overall functionality through the coating process [61].

Oral ulcers are a common form of mucosal lesion, impacting over 20% of the population, and profoundly affecting patients’ quality of life by causing intense pain and compromising the integrity of the mucosal barrier. This damage makes underlying tissues vulnerable to mechanical irritation and bacterial invasion. Conventional treatments, such as mouthwashes, sprays, and ointments, are often washed away by saliva or displaced by food during chewing, preventing them from providing lasting protection. As a result, an ideal therapeutic patch should exhibit strong adhesion to tissue, long-term retention in the moist oral cavity, excellent adaptability to the tissue, and protective surface properties [62,63].

In response to these challenges, Zhang et al. introduced a novel Janus hydrogel patch, ANSB, inspired by the asymmetric, multilayered structure of oral mucosa. This hydrogel consists of two layers: an inner adhesive layer and an outer lubricating layer. The adhesive inner layer is crafted from a prepolymer solution containing gelatin, acrylic acid (AA), N-hydroxysuccinimide ester (NHS), and a photoinitiator (Irgacure-2959), which is poured into molds and initially polymerized under 365 nm ultraviolet light. The interaction between amine groups on the tissue surface and the prepolymer forms a strong, covalent bond that ensures rapid, durable adhesion. After the initial polymerization, a second prepolymer mixture—comprising N-acryloylglycinamide (NAGA) and sulfobetaine methacrylate (SBMA)—is added to the mold and polymerized once more under ultraviolet light. This process allows the unreacted double bonds on the adhesive layer to covalently cross-link with the SBMA and NAGA monomers, effectively integrating the lubricative and adhesive layers into a unified structure. This dual-layer design offers significant protection against bacterial infiltration and food residue. In comparison to commercially available alternatives, the ANSB hydrogel patch exhibits superior adhesive strength and extended retention time, greatly enhancing the healing process of oral ulcers and confirming its therapeutic potential [64].

Perforating oral fistulas (POFs) represent a frequent and serious complication following reconstructive surgery of the oral or pharyngeal regions. These fistulas are notoriously difficult to heal due to factors such as the moist environment, contamination by saliva, and the dynamic mechanical forces exerted during activities like chewing and swallowing. Traditional treatments, including iodine gauze dressings and negative pressure wound therapy, have demonstrated limited effectiveness, highlighting the pressing need for novel biomaterials capable of providing sustained sealing and fostering tissue regeneration [65,66].

Ju et al. introduced a cutting-edge multifunctional Janus adhesive, designed with a mechanical support layer (SL) and a repair layer (RL), offering an innovative solution for effectively sealing and repairing perforating fistulas. The surface enzyme-initiated polymerization (SEIP) process is employed, wherein glucose and glucose oxidase (GOx) catalyze the production of hydrogen peroxide (H_2_O_2_), initiating free radical polymerization. This process enables the adhesive to capture moisture and blood at the surface, forming a seamless interface. The SL, composed of polyacrylic acid (PAA) crosslinked with chondroitin sulfate (CS), provides enhanced mechanical strength and energy dissipation. The RL, made from a combination of CSMA, DMAA, and AAC-NHS, covalently binds to tissue surface amino groups through NHS ester linkages, ensuring robust adhesion in the moist oral cavity. The H_2_O_2_ generated by GOx not only inhibits the growth of pathogens such as *Staphylococcus aureus* and *Escherichia coli* but also achieves a remarkable antimicrobial efficacy of 99%. Additionally, Fe[Gly]_2_ serves as a potent scavenger of reactive oxygen species (ROS), reducing oxidative stress and shielding cells from damage. This material also facilitates macrophage polarization from the pro-inflammatory M1 phenotype to the anti-inflammatory M2 phenotype, thereby lowering levels of inflammatory cytokines such as IL-6 and TNF-α. Notably, the biocompatibility of this material is exceptional, with biochemical assays and organ histological analysis showing no signs of toxicity. Furthermore, RNA sequencing of the JHA groups revealed a marked inhibition of the JAK/STAT and IL-17 signaling pathways, resulting in reduced inflammation and fibrosis [23].

## 4. Orthopedic and Musculoskeletal Reconstruction

In the realm of orthopedic and musculoskeletal therapies, Janus hydrogels play a crucial role in promoting the healing of injured bones and tissues, reducing inflammation, and delivering vital support throughout the recovery phase [67,68]. Their unique design enhances their multifunctionality, fostering superior tissue regeneration while simultaneously minimizing complications. As a result, Janus hydrogels are emerging as a highly promising material for addressing fractures, ligament damage, and soft tissue defects, offering significant potential for advanced reparative treatments [69,70,71].

**Figure 5:**
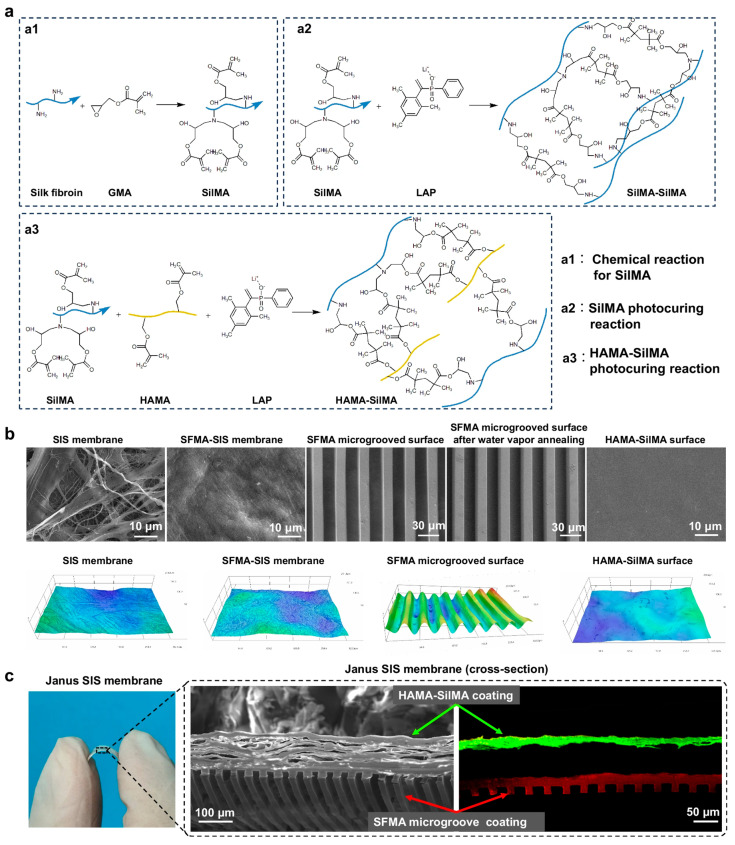
Application of Janus hydrogels in orthopedic and musculoskeletal reconstruction. (**a**) SF chemically modified by glycidyl methacrylate (SilMA) and the photocuring reactions of SilMA and HAMA-SilMA. (**b**) SEM and 3D optical images of the SIS, SFMA-SIS, SFMA microgroove coating without water vapor annealing, SFMA microgroove coating after water vapor annealing, and HAMA-SilMA coating. (**c**) SEM and CLSM cross-section images of Janus SIS membrane. Red arrow indicates the SFMA microgroove coating; Green arrow indicates the HAMA-SilMA coating. Copyright 2025 Springer Nature [72].

Bi et al. developed a Janus-structured decellularized membrane with anisotropic cellular guidance and anti-adhesion properties for postsurgical dural repair, addressing the critical clinical challenge of cerebrospinal fluid leakage and epidural fibrosis following spinal interventions (Figure 5). Surgical management of spinal pathologies, while indispensable, remains inherently complex with complication rates of 1–17% for dural tears in lumbar procedures, complications that frequently progress to inflammatory cascades, fibroblast hyperproliferation, and recalcitrant epidural adhesions complicating revision surgeries [72].

The team engineered a bilayered small intestinal submucosa (SIS)-based construct through silk fibroin hydrogel modifications, creating an extracellular matrix (ECM)-mimetic Janus membrane with dual functionality. This biomaterial combines SIS’s native porous fibrous architecture—providing three-dimensional cellular infiltration capacity—with stratified functional coatings: An inner SFMA microgrooved layer (20 µm channels) of methacrylated silk fibroin (SiMA) promotes fibroblast alignment and anti-inflammatory macrophage polarization through topographical guidance while facilitating ECM remodeling via vapor-annealed structural stabilization. The outer HAMA-SiMA barrier layer, comprising hyaluronic acid methacrylate (HAMA) and SiMA, exhibits protein-repellent characteristics and prevents peridural tissue adhesion through optimized surface energetics.

Photocuring and micromolding techniques enabled precise spatial control over layer integration, achieving strong interfacial bonding through covalent/mechanical interlocking. In rodent dural defect models, the Janus membrane demonstrated synergistic biological effects, such as directional ECM reconstruction mimicking native dura microstructure combined with significant fibrosis reduction (*p* < 0.01 vs. controls). This biomimetic approach leverages SIS’s inherent bioactivity while introducing engineered anisotropy—a paradigm shift from conventional isotropic barriers—offering dual-phase therapeutic action critical for preventing postoperative complications while supporting tissue regeneration. The technology presents transformative potential for spinal surgery applications requiring simultaneous defect sealing and adhesion prevention.

Pan et al. successfully engineered a multifunctional Janus membrane featuring an asymmetric functional gradient, utilizing a sophisticated multilayer self-assembly process. The upper GBR-A layer consists of a hydrophobic PCL coating, while the lower GBR-B layer integrates hydrophilic HAn, PCL, and PEG components. The GBR-A layer, characterized by its smooth and dense structure, acts as a hydrophobic barrier, effectively preventing cell adhesion and infiltration by surrounding soft tissues. In contrast, the GBR-B layer is rough, porous, and hydrophilic, fostering cell attachment, proliferation, and osteogenic differentiation [73].

This Janus membrane not only demonstrates superior mechanical properties but also offers a controlled degradation rate, ensuring reliable spatial stability throughout the bone healing and regeneration process. In an in vivo rabbit femoral defect model, the GBR membrane provided stable fixation for bone graft materials, significantly stimulating the formation of new bone tissue.

Tendon injuries are a common complication associated with fractures, often hindering the recovery process of patients. While some commercially available materials offer certain therapeutic benefits in specific cases, the mechanical strength of most is inadequate, failing to meet the high-strength support required during tendon repair. These materials struggle to effectively replicate the physical properties of tendon tissue, resulting in suboptimal healing outcomes [74].

Freedman et al. developed the Janus Tough Adhesive (JTA) hydrogel, which showcases outstanding biocompatibility, mechanical properties, and drug delivery capabilities, offering substantial promise for clinical application. Composed of FDA-approved materials such as alginate, chitosan, and polyacrylamide—widely utilized in clinical settings—the hydrogel ensures both safety and effectiveness. Its innovative dual-sided design incorporates a robust, dissipative matrix on one side and a highly adhesive surface on the other. This configuration not only provides significant mechanical load-bearing capacity but also ensures strong adhesion, making it particularly beneficial for repairing dynamic tissues like tendons. By offering stable support during tissue movement, the JTA hydrogel effectively mitigates the risk of mechanical failure or degradation, a common issue with traditional materials. The hydrogel demonstrates an impressive tensile strength of around 2.5 MPa, far exceeding the typical range of 0.5–1 MPa for conventional hydrogels, showcasing its superior capacity to withstand stretching forces. Furthermore, its elongation at a break of up to 180% allows it to endure substantial deformation without rupture, making it suitable for high-load, dynamic environments. With an adhesion strength surpassing 1000 J/m^2^, the hydrogel securely bonds to damaged tendons and other tissues, preventing detachment or displacement during stretching or sliding. In the study, the pharmacological activity of the JTA hydrogel was validated through the controlled release of the corticosteroid triamcinolone acetonide (CORT). The results demonstrated significant anti-inflammatory effects, including feedback inhibition of endogenous cortisol levels within two days of implantation. Moreover, a decrease in serum GROα levels, a chemokine responsible for neutrophil recruitment and activation, further confirmed the drug’s effectiveness. Additionally, an increase in CD146-positive cells, a marker of tendon stem/progenitor cells, suggests the potential of the JTA hydrogel in promoting tendon regeneration and enhancing healing processes [75].

## 5. Intelligent Functionalized Systems

Janus hydrogels, with their unique dual-sided structure, provide enhanced functionality in targeted drug release, enabling controlled and sustained therapeutic effects. Additionally, their versatility extends to sensor applications, where they serve as responsive materials capable of detecting specific stimuli, offering promising prospects in diagnostic and monitoring systems. Multifunctional Janus hydrogels offer substantial advantages over uniformly functionalized hydrogels in MRI-based imaging applications. By segregating functionalities into discrete hydrogel domains, Janus architectures significantly enhance the localized loading of MRI contrast agents, such as the targeted concentration of magnetic nanoparticles on one side, thereby improving imaging performance. This spatial organization further enables dual-modality imaging within a single platform by incorporating an MRI contrast agent on one face and an optical or X-ray contrast agent on the other, resulting in a bifunctional probe capable of visualization across multiple imaging modalities. Critically, the spatial separation between functional layers minimizes mutual interference or quenching effects among contrast agents and allows for precise spatial control, ensuring that each compartment achieves its intended functionality—such as optimized contrast enhancement or targeted biological interactions—with maximal efficiency. Through this integrated and anisotropic design, Janus hydrogels attain levels of contrast enhancement and multimodal imaging capability that are unattainable with conventional single-phase hydrogels [76,77].

### 5.1. Integrated Diagnosis and Treatment Design

Wang et al. engineered a multifunctional Janus nanofiber patch (J-NFP) that seamlessly combines anti-adhesion and wound-healing properties, utilizing a blend of tissue surface engineering and nanozyme technology, specifically targeting uterine injuries and superficial tissue repair. Employing a sequential electrospinning technique, initiators and cerium oxide nanoparticles (CeNPs) were strategically incorporated into separate fibrous layers. Following this, surface-initiated atom transfer radical polymerization (SSI-ATRP) was utilized to graft zwitterionic polymer brushes, such as poly(sulfobetaine methacrylate) (PSBMA), onto individual nanofibers, creating a surface with remarkable lubricity. The CeNP-infused patch effectively decomposes hydrogen peroxide (H_2_O_2_), releasing oxygen to alleviate oxidative stress and inflammation at the site. In vitro experiments revealed that the Ce@NFP patch reduced intracellular reactive oxygen species (ROS) levels and promoted the expression of the anti-inflammatory cytokine IL-10. Additionally, the PSBMA brush-grafted upper surface maintained its fibrous structure, significantly limiting the adhesion of proteins, platelets, bacteria, and cells. This innovative patch demonstrates broad applicability for both external and internal soft tissue repair, holding great potential for future clinical use [78].

**Figure 6:**
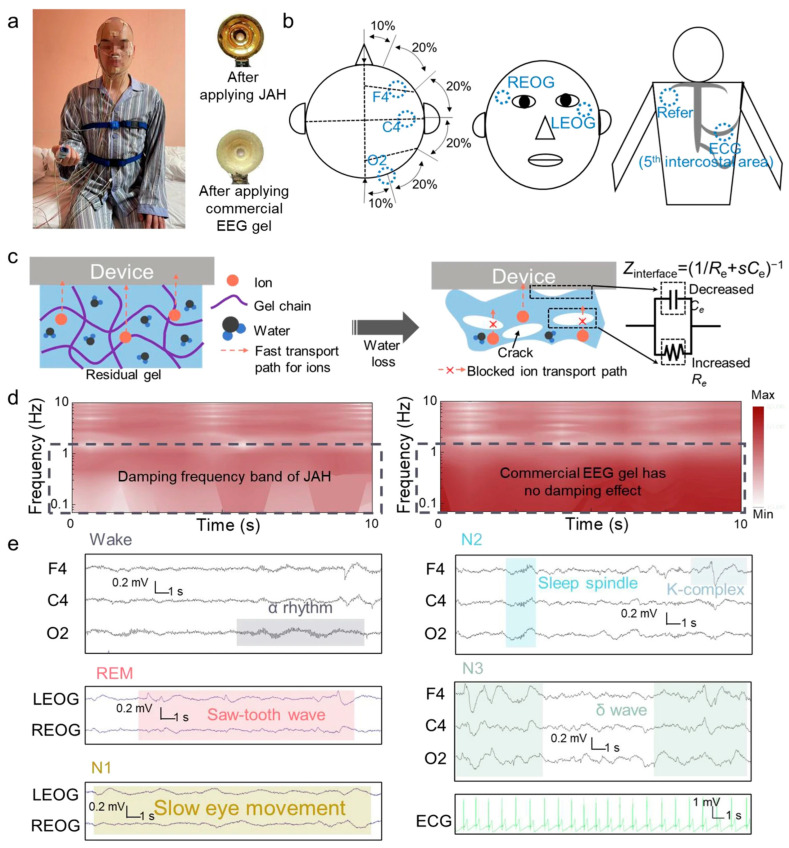
Application of Janus hydrogels in integrated diagnosis and treatment design. (**a**) Clinical sleep monitoring and the difference in device electrodes after using JAH and commercial EEG gel. (**b**) The positions of JAH for EEG, EOG, and ECG during clinical sleep monitoring. (**c**) Effect of residual gel on bioelectrical signals. (**d**) Damping effect of JAH on dynamic noise in the frequency range 0.1–1 Hz. (**e**) Raw EEG, EOG, and ECG data measured from the polysomnography (PSG) system (F4, C4, O2, LEO, and REOG) by JAH, capturing the characteristic signals of the five sleep stages (awake, N1, N2, N3, and REM). Copyright 2024 Springer Nature [79].

Luo et al. introduce an asymmetric adhesive hydrogel biointerface (JAH) designed to address longstanding limitations in physiological signal monitoring, specifically, the dual obstacles of motion-induced signal distortion and interfacial residue accumulation during electrophysiological recordings (Figure 6). This innovation enables frequency-specific noise suppression, artifact-resistant signal transduction, and clean-device detachment, advancing noninvasive diagnostic paradigms [79].

Bioelectrical signatures—including electrocardiograms (ECG), electroencephalograms (EEG), and auditory brainstem responses (ABR)—form essential diagnostic biomarkers for disorders ranging from arrhythmias to neurological deficits. However, their interpretative value is critically undermined by low-frequency artifacts (0.1–1 Hz) generated during resting respiration, which induce waveform aberrations that mimic pathological patterns and obscure true physiological states.

The JAH system integrates a dual-phase polymer network comprising acrylamide copolymers solvated in ethylene glycol–aqueous electrolytes, with sodium chloride optimizing ionic mobility and silver nanostructures enhancing interfacial charge transport. Fabrication via gradient-controlled radical polymerization and nanoparticle deposition yields a Janus material exhibiting spatially modulated functionalities: directional viscoelastic damping (achieving >90% attenuation of 0.2–1 Hz interference) coexists with pressure-adaptive adhesion (≈15 kPa interfacial bonding strength). In auditory pathophysiology models, JAH outperformed percutaneous electrodes in ABR detection sensitivity, resolving signals at 25 dB (healthy subjects) and 35 dB (otitis media models) while maintaining waveform integrity against thoracic motion artifacts. Polysomnographic evaluations revealed 92% concordance with standard scalp leads in tracking sleep architecture oscillations (α, spindle, and δ waves), demonstrating particular efficacy in detecting apnea-related spectral shifts.

This platform’s breakthrough lies in its dual-mode operation: frequency-dependent viscoelastic damping mechanically isolates biosensors from sub-Hertz physiological motions, while redox-triggered adhesion permits nondestructive device removal. The hydrogel’s tunable viscoelastic spectrum (a storage modulus spanning four orders of magnitude) facilitates both conformal tissue coupling and inertial decoupling from low-frequency displacements. By contrast with conventional adhesives that either sacrifice signal fidelity for strong bonding or prioritize detachment at the expense of motion rejection, JAH’s orthogonally engineered properties enable simultaneous high-resolution biosensing and tissue-protective operation, a critical advancement for chronic monitoring applications requiring repeated sensor redeployment.

Chen et al. engineered an implantable colloidal crystal hydrogel catheter integrated with resistive strain sensors for real-time intestinal pressure monitoring, addressing critical challenges in gastrointestinal health surveillance through biomimetic sensing integration. This stratified architecture features the following: (1) an outermost biocompatible hydrogel layer of poly(N-isopropylacrylamide-co-bis-acrylamide) [P(NiPAAm-bis-AA)] exhibiting hydrophilic inverse opal architecture that demonstrates dual-mode responsiveness—resistive variations (ΔR/R) and structural color transitions (e.g., blue→green)—under mechanical, thermal, and ionic stimuli; (2) a middle functional stratum combining constantan foil strain-sensitive elements with polydimethylsiloxane (PDMS) encapsulation for robust signal conduction and electromechanical protection; and (3) a flexible PDMS tubular substrate providing structural integrity during peristaltic deformation.

The system mimics intestinal mechanotransduction networks by synergizing biocompatible hydrogel matrices, optical–electrical dual-signal transduction, and tissue-conformal adhesion, achieving the seamless integration of mechanosensation, signal transduction, and output. This bioinspired design overcomes traditional limitations in chronic GI monitoring through its self-adherent properties (≈12 kPa interfacial adhesion energy), millimeter-scale flexibility (bending radius < 3 mm), and real-time pressure resolution (0.2 kPa sensitivity). The technology establishes new paradigms for closed-loop diagnostic systems by enabling continuous luminal pressure mapping correlated with motility disorders, while its chromatic response provides visual feedback complementary to electronic readouts [80].

Wang et al. developed a dual-layer epidermal interface with autonomous adhesion that maintains structural integrity under 20% mechanical stretching and prolonged artificial sweat immersion (24 h), preserving continuous skin contact for over 7.5 h without supplemental adhesives, a critical capability for monitoring dynamic cutaneous biomarkers. This mechanical resilience enables reliable data acquisition during physical activity, where conventional sensors typically fail due to motion artifacts or delamination [81].

The sub 2 μm heterostructure incorporates two functionally optimized strata. A van der Waals-interactive hydrophobic base layer of PU-PDMS with 10 μm micropores achieves skin-conformal adhesion (78.5 ± 8.0 μJ/cm^2^) through molecular-scale surface interactions, while its porosity enables vapor transmission rates matching unhindered atmospheric exchange (≈85% relative humidity maintenance). Complementing this, a hydrophilic PVA-PAA nanofiber mesh demonstrates rapid interstitial fluid uptake (0.8 μL/s) via programmable hydrogen-bond networks that swell reversibly without structural degradation, addressing the critical challenge of sweat accumulation in continuous monitoring scenarios. This material’s innovation pioneers a new paradigm in epidermal biosensing by harmonizing three historically conflicting properties: robust biotic–abiotic interfacial coupling, unimpeded transepidermal water loss, and active fluid management. Early validation trials demonstrate that millipH–unit resolution in tracking skin surface acidity fluctuations correlated with inflammatory conditions, though comprehensive multicenter trials must evaluate cross-demographic variability in adhesion kinetics and long-term dermal biocompatibility.

The Janus architecture’s breakthrough lies in its biomimetic design philosophy. Rather than forcing material compromises, it spatially decouples antagonistic functionalities. Mechanical compliance and chemical sensing are allocated to separate strata operating synergistically through interfacial charge transfer. This departure from conventional homogeneous sensor designs resolves the persistent wearable technology paradox, where enhanced sensor fidelity typically exacerbates skin irritation, positioning the platform as a viable solution for chronic disease management through unobtrusive physiological surveillance.

Cervical health is integral to a woman’s overall well-being, particularly regarding fertility and gynecological disorders such as cervical cancer. Monitoring pressure changes within the cervix in real time provides a powerful tool for the early detection of abnormalities, enabling timely intervention and preventing serious complications. Traditional monitoring methods, including ultrasound and manual examinations, often demand complex equipment and specialized expertise, and they fail to offer continuous, real-time data. Moreover, these approaches can cause discomfort and are typically associated with high costs.

Chen et al. introduced a flexible, highly sensitive, and cost-efficient Janus-wetting biomimetic hydrogel pressure sensor, specifically designed for cervical health monitoring. Drawing inspiration from the Janus-wetting behavior of lotus leaves, the sensor incorporates tunable structural colors and selective adhesion properties. The system comprises a flexible, hollow cylindrical polydimethylsiloxane (PDMS) substrate, four pressure sensors for electrical signal transmission, and a P(NiPAAm-bis-AA) hydrogel membrane scaffold, which displays Janus characteristics and undergoes structural color changes. This hydrogel scaffold is primarily composed of N-isopropylacrylamide (NiPAAm) and bis-acrylamide (bis-AA) copolymers. The thermoresponsive NiPAAm monomer imparts temperature sensitivity to the hydrogel, while bis-AA enhances the material’s mechanical strength and stability. To improve electrical conductivity, reduced graphene oxide (rGO) is integrated into the hydrogel, facilitating the conversion of pressure variations into electrical signals.

The P(NiPAAm-bis-AA) hydrogel scaffold features an anti-proteinaceous structure, enabling it to generate structural color shifts in response to changes in its internal nanostructure. Thanks to its Janus-wetting design, the sensor adheres securely to the cervical wall yet can be easily removed, significantly reducing patient discomfort during use [82].

### 5.2. Targeted Delivery and Barrier Penetration

Janus hydrogels, unlike traditional hydrogels, possess a unique dual-sided structure with hydrophilic and hydrophobic faces, allowing precise targeting in drug release. The hydrophilic side efficiently adsorbs and regulates drug release, while the hydrophobic side prevents premature release, ensuring sustained and accurate delivery [83]. By adjusting the ratio of hydrophilic to hydrophobic surfaces and the degree of crosslinking, the physicochemical properties of Janus hydrogels can be optimized to control the release rate and pattern. This tunability offers a distinct advantage over traditional hydrogels, particularly in targeted drug delivery and precision therapy [84,85,86,87].

**Figure 7:**
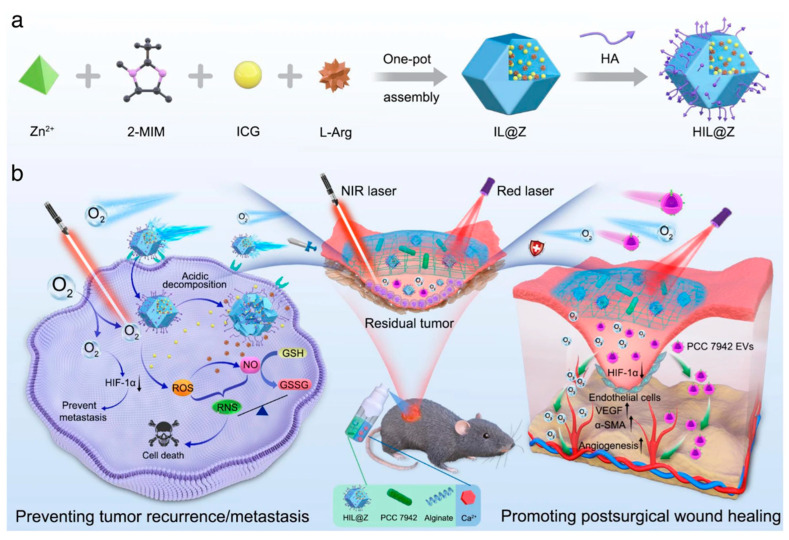
Application of Janus targeted delivery and barrier penetration. (**a**) Preparation of HIL@Z nanodrug. (**b**) Schematic showing the in situ formation and action mechanism of sprayed HIL@Z/P/H containing HIL@Z nanodrug and PCC 7942 within the postsurgical wound bed. Under red laser irradiation, HIL@Z/P/H produces abundant O_2_ through photosynthesis and effectively relieves the hypoxia microenvironment. In tumor cells, the intracellular cascade reactions induced by HIL@Z nanodrug generate plentiful reactive species (ROS, NO, and RNS) and lower the GSH level, accompanied by significant HIF-1α downregulation with the aid of O_2_, resulting in effective inhibition of residual tumor recurrence/metastasis. Within the postsurgical wound, the excessively generated O_2_ and PCC 7942-secreted EVs accelerate the wound healing process by downregulating HIF-1α expression and upregulating VEGF level. Copyright 2024 Springer Nature [89].

Melanoma, the deadliest and most metastatic form of skin cancer, remains a major therapeutic challenge. Surgery is the primary treatment method; however, incomplete tumor excision, coupled with recurrence and metastasis, poses a significant threat to patient survival. Moreover, the immense pain and suffering caused by large, unhealed postoperative wounds continue to complicate recovery [83,88]. Chen et al. developed a novel hydrogel designed to address two critical issues following malignant melanoma surgery: tumor recurrence/metastasis and delayed wound healing, exhibiting Janus-like functional properties (Figure 7). The researchers created a sprayable therapeutic hydrogel (HIL@Z/P/H), which encapsulates tumor-targeting nanomedicines and photosynthetic cyanobacteria (PCC 7942). The hyaluronic acid-modified nanomedicine consists of indocyanine green (ICG) and L-arginine (L-Arg), loaded into a zeolitic imidazolate framework (ZIF-8) and coated with hyaluronic acid (HA). The hydrogel utilizes photodynamic therapy (PDT) to induce a cascade reaction that disrupts cellular redox homeostasis by increasing intracellular active species and reducing GSH levels. This results in the production of reactive oxygen species (ROS), nitric oxide (NO), and reactive nitrogen species (RNS), leading to the destruction of tumor cells. Under prolonged near-infrared (NIR) laser exposure, PCC 7942 continuously generates oxygen via photosynthesis, effectively reversing the hypoxic microenvironment of both tumor cells and postoperative wounds. This supplementary oxygen supply not only enhances the efficacy of PDT but also induces cell death triggered by nitrosative stress, preventing the local recurrence of residual tumor cells. Furthermore, it inhibits the hypoxia-inducible factor 1α (HIF-1α) signaling pathway, promoting angiogenesis, accelerating wound healing, and suppressing distant metastasis of tumor cells. Although structurally homogeneous, the hydrogel’s dual functionality is achieved through the synergistic effects of the tumor-targeting nanomedicine and photosynthetic cyanobacteria, highlighting the potential of Janus-like hydrogels in postoperative cancer treatment [89].

Studies show that the permeability of the oral mucosa is vastly superior to that of the skin, being 4 to 4000 times greater, which significantly enhances drug absorption and delivery efficiency. You et al. exploited the unidirectional diffusion properties of Janus materials, enabling targeted drug delivery while preventing premature release due to mucosal fluids. This directional diffusion not only optimizes drug delivery but also minimizes waste.

In this research, the hydrogel formulation consists of four key components: poly(methyl vinyl ether maleic anhydride) (Gantrez), hydroxymethyl cellulose (Natrosol), Kollidon VA (a copolymer of vinylpyrrolidone and vinyl acetate), and 2-hydroxypropyl-β-cyclodextrin (HPCD). Initially, a polyester fabric is coated with the hydrophobic polymer PHFDMA through the iCVD technique, forming a uniform hydrophobic layer that blocks moisture infiltration and maintains the stability of the drug. The coated fabric is then subjected to a strong base solution, which selectively hydrolyzes one side of the fabric to create a hydrophilic surface, while the other side retains its hydrophobic properties. This dual-sided structure of the Janus patch allows one side to absorb moisture, while the other remains impermeable [14].

The hydrogel, enriched with resveratrol, is applied to the hydrophilic surface of the Janus patch. When this patch is placed on the oral mucosa, it bypasses the first-pass metabolism that typically occurs with oral administration. In vitro pharmacokinetic analysis demonstrates enhanced resveratrol absorption through murine skin with the Janus patch. Given the high permeability of the oral mucosa, even more favorable absorption outcomes are expected in vivo.

Wang et al. designed a bilayer Janus bandage with distinct wettability characteristics tailored for wound care, offering targeted drug delivery and multifunctional therapeutic effects. The inner hydrogel layer of the bandage encapsulates and slowly releases drugs in a unidirectional manner directly at the wound site. This layer also provides antibacterial properties, promotes biocompatibility, and supports hemostasis, thereby enhancing the healing process. The outer hydrophobic layer is formed by submerging a standard dressing in an OTS solution, which creates a hydrophobic coating. The inner layer combines chitosan or HACC with PVA and PAA, which work synergistically to improve the dressing’s absorbency, softness, controlled drug release, and adaptability to the wound, optimizing both care and delivery of the therapeutic agents. Chitosan contributes to enhanced biocompatibility and antimicrobial activity, while HACC offers superior antibacterial performance, making it ideal for wound-healing environments. The hydrophilic Janus bandage, known for its softness and elasticity, adapts well to various wound surfaces, including those in curved or mobile areas, reducing secondary injuries and minimizing patient discomfort. Despite its multifunctionality, further enhancements in mechanical strength and production costs are essential to effectively address larger or deeper wounds [90].

Nummelin et al. engineered a Janus dendrimer that spontaneously forms hydrogels with remarkable mechanical strength tailored for the controlled release of therapeutic agents. The dendrimer’s hydrophilic and hydrophobic components are connected through “click chemistry”, utilizing hydroxyl-functionalized, hydrophilic bis–MPA as the core framework and integrating hydrophobic alkyl chains to create the amphiphilic Janus structure. The synthesis process involves the reaction of azide-functionalized dendron monomers with alkyne-modified bis-MPA monomers in the presence of a Cu(II)/sodium ascorbate catalytic system. Following this, column chromatography is employed for purification, yielding the desired Janus dendrimers. By modifying the positions of the alkyl chains (e.g., at the 3.4; 3.5; or 3, 4, 5 positions), dendrimers with varied structural forms are synthesized. This hydrogel platform enables the controlled release of various drug types—including small molecules, peptides, and proteins—with release rates determined by the molecular weight. Smaller molecules are released more quickly, while larger molecules, such as enzymes, are released more gradually, maintaining their bioactivity throughout the process [91].

## 6. Conclusions and Future Work and Challenges

Janus hydrogels have rapidly evolved into a class of multifunctional biomaterials, achieving functional properties through asymmetric structural designs that are unattainable with traditional single-phase hydrogels. Innovative fabrication strategies now enable precise control over the composition and properties of each side of the hydrogel (Table 1). For instance, bioadhesive Janus hydrogels have been developed to seamlessly integrate with tissues on one side while exhibiting anti-adhesive and biofouling-resistant characteristics on the other. These advancements highlight the vast potential of Janus designs: researchers have successfully integrated incompatible functions—such as insulating/conductive and adhesive/anti-adhesive properties—into a single platform, significantly expanding the performance boundaries of flexible electronic devices, sensors, and biomedical interfaces. Currently, Janus hydrogels are being explored in diverse applications, including tissue repair, postoperative anti-adhesion barriers, intelligent drug delivery, hemostatic dressings, and motion sensors, demonstrating their unique advantages in addressing complex biomedical challenges.

Despite this progress, several challenges must be overcome to fully realize the potential of Janus hydrogels. First, current fabrication methods are often complex, multi-step processes with relatively high cost and time demands. Developing more economical and scalable manufacturing techniques is critical to improving production efficiency and reproducibility so that Janus hydrogels can transition from the lab bench to commercial and clinical scales. Second, maintaining the interface stability between the two distinct hydrogel phases remains a concern. Many Janus constructs rely on dynamic bonding or physical entanglement to join dissimilar layers, which can be prone to bond weakening or delamination over time. Finally, the long-term biocompatibility and in vivo performance of Janus hydrogels must be thoroughly validated. The introduction of novel chemistries means that biodegradation behavior and any chronic immune responses need close evaluation. Ensuring that Janus hydrogels remain non-toxic, do not provoke inflammation, and retain functional stability over extended implantation or use periods is essential for clinical acceptance. By addressing these challenges—improving scalable fabrication, strengthening interface and bulk mechanical integrity, and verifying long-term bio-safety—researchers can pave the way for Janus hydrogels to transition from an innovative concept to a practical reality in biomedical and engineering applications. The continued convergence of materials science, bioengineering, and microfabrication technologies will be key to surmounting the current limitations and unlocking the next generation of smart Janus hydrogel systems.

## Figures and Tables

**Table 1:** Summary of Janus hydrogels for biomedical applications.

Key Materials	Construction Strategy	Application Function	Ref.
SFMA microgroove coating, HAMA-SilMA anti-adhesion coating, SIS substrate	UV photocuring and micromolding	Spinal dura mater repair and anti-adhesion barrier	[72]
Alginate, polyacrylamide, chitosan, and triamcinolone acetonide delivery	Double-network formation and unilateral adhesion	Tendon adhesion enhancement and anti-inflammatory therapy	[75]
HAD polymer (hyaluronic acid–dopamine–methacrylate), LAP photoinitiator	Injectable UV photocrosslinking and asymmetric adhesion	Sutureless gastric perforation repair and anti-adhesion barrier	[42]
CPAMC hydrogel and PCA hydrogel	Interfacial copolymerization of redox-responsive conductive and anti-adhesion hydrogels	Cardiac infarction repair and prevention of postoperative tissue synechia	[45]
Acrylamide hydrogel with silver nanoparticles	Natural sedimentation-assisted gelation with asymmetric silver nanoparticle distribution	Selective respiration noise damping and stable bioelectrical signal transmission	[79]
PEGDA-BTO-Au hydrogel and GelMA-VEGF hydrogel	Extrusion-based 3D printing of dual-functional hydrogel layers	Sonodynamic bacterial elimination and programmable wound healing	[92]
Strontium–apatite-mineralized collagen, PCLMA dense layer	Biomimetic mineralization and photocrosslinking	Guided bone regeneration and anti-soft tissue invasion	[57]

## Data Availability

No new data were created or analyzed in this study. Data sharing is not applicable to this article.
